# Poor survival in stage IIB/C (T4N0) compared to stage IIIA (T1-2 N1, T1N2a) colon cancer persists even after adjusting for adequate lymph nodes retrieved and receipt of adjuvant chemotherapy

**DOI:** 10.1186/s12885-016-2446-3

**Published:** 2016-07-13

**Authors:** Quyen D. Chu, Meijiao Zhou, Kaelen L. Medeiros, Prakash Peddi, Mindie Kavanaugh, Xiao-Cheng Wu

**Affiliations:** Department of Surgery, Louisiana State University Health Sciences Center, Shreveport, Louisiana 71130 USA; Department of Medicine, Division of Hematology and Oncology, Louisiana State University Health Sciences Center, Shreveport, Louisiana 71130 USA; Feist-Weiller Cancer Center, Shreveport, Louisiana 71130 USA; Louisiana Tumor Registry and Epidemiology, New Orleans, Louisiana 71102 USA; School of Public Health, Louisiana State University Health Sciences Center, New Orleans, Louisiana 71102 USA

**Keywords:** Colon cancer, Stage IIB/C colon cancer, Stage IIIA colon cancer

## Abstract

**Background:**

A survival paradox between Stage IIB/C and Stage IIIA colon cancers exists. It is unclear how adequate lymph nodes dissection (LN) and post-surgery chemotherapy contribute to the survival paradox. We intended to assess the impact of these two factors on the survival paradox.

**Results:**

We evaluated 34,999 patients diagnosed with stage IIIA or stage IIB/C colon cancer in 2003–2012 from the National Cancer Data Base. The 5-year overall survival (OS) was 73.5 % for stage IIIA and 51.1 % for stage IIB/C (*P* < 0.0001). The 5-year OS was 84.1 % for stage IIIA with post-surgery chemotherapy, 70.8 % for stage IIB/C with ≥ 12 LNs retrieved with chemotherapy, 53.9 % for stage IIB/C < 12 LNs with chemotherapy, 49.5 % for stage IIIA without chemotherapy, 43.7 % for stage IIB/C ≥ 12 LNs retrieved without chemotherapy, to 27.7 % for stage IIB/C < 12 LNs without chemotherapy. Even among stage IIB/C who had optimal treatment (≥12 LNs retrieved, received chemotherapy), OS remains lower than stage IIIA with chemotherapy. After adjusting LN dissection and chemotherapy in addition to the adjustment of other clinical factors, the survival paradox was reduced from HR = 1.76 (95 % CI: 1.68–1.85) to HR 1.51 (95 % CI: 1.44–1.59).

**Conclusions:**

LN dissection and post-surgery chemotherapy partially explained the survival paradox. More research is warranted to identify other factors that contribute to this paradox. Future iteration of TNM staging system should take this into consideration.

## Background

For most solid cancers, the 7^th^ edition of the American Joint Committee on Cancer (AJCC) TNM staging system accurately prognosticates outcome with lower stage cancers having better prognosis than higher stage cancers [1]. However, colon cancer is one of the few exceptions. For stage IIB/C and stage IIIA, there exists a survival paradox [2–5]; the 5-year overall survival for patients with stage IIIA is approximately 70 % versus 46–61 % for stage IIB/C [1]. Such a paradox is attributed to several factors according to previous studies, such as stage migration due to inadequate nodal sampling or lack of systemic therapy for stage IIB/C [2, 6]. We hypothesize that stage IIB/C is inherently more aggressive than stage IIIA, even after adjusting for receipt of chemotherapy and adequate nodal sampling. We propose to assess the simultaneous contribution of lymph node dissection and receipt of post-surgery chemotherapy to this survival paradox.

## Methods

### Data Source

The nationally recognized National Cancer Data Base (NCDB) is a joint project of the Commission of Cancer (CoC) of the American College of Surgeons and the American Cancer Society. More than 1500 CoC-accredited facilities in the U.S. contribute clinical information to the database. Approximately 70 % of newly diagnosed cancer cases in the U.S and 30 million historical records are captured in the database (https://www.facs.org/quality-programs/cancer/ncdb/puf). The data in the Participant User File (PUF) were de-identified and in compliance with the privacy requirements of the Health Insurance Portability and Accountability Act (HIPAA). The study was exempted from Institutional Review Board (IRB) approval by the Louisiana State University Health Sciences Center-Shreveport.

### Study population

A cohort of 34,999 cases of stage IIIA or stage IIB/C colon cancer cases (ICD-0-3; C18.0, C18.2 to C.18.9) diagnosed in 2003–2012 in the NCDB were analyzed to determine significant factors associated with 5-year overall survival (OS). Patients were staged based on the 6^th^ and 7^th^ edition of the AJCC/TNM staging system [1]. Patients were further divided into six subgroups based on number of lymph nodes (LNs) dissected and status of chemotherapy use: (1) Stage IIIA + chemotherapy, (2) Stage IIB/C, ≥ 12 LNs + chemotherapy, (3) Stage IIB/C, < 12 LNs + chemotherapy, (4) Stage IIIA, no chemotherapy, (5) Stage IIB/C, ≥ 12 LNs, no chemotherapy, and (6) Stage IIB/C, < 12 LNs, no chemotherapy.

According to the NCDB’s PUF dictionary [7], comorbidity was reported as Charlson/Deyo score: 0, 1 or 2 [8, 9]. Age at diagnosis, race, facility type, facility location, urban/rural, insurance status, income and education levels for each patient’s area of residence, comorbid conditions, anatomic site, tumor grade, surgical margin status, chemotherapy, and number of lymph nodes retrieved were variables selected for evaluation. NCDB does not have information on cause-specific survival and therefore, overall survival was calculated based on death from all causes.

### Statistical Analysis

We calculated the propensity scores by stage IIB/C and stage IIIA using a multivariable logistic regression model. Only cases with matched scores based on potential confounders (i.e., age, race, distance from cancer reporting facility, facility type, facility location, rural/urban, insurance, income, education, comorbidity, primary site, grade, and surgical margins) were included in the multivariable analysis. The purpose of this approach was to ensure that stage IIB/C and stage IIIA cases were comparable to reduce potential confounder effect. Descriptive statistics for the different variable were presented. Univariable analysis of each variable was performed using chi-square test for categorical data and ANOVA for numerical data. The Kaplan-Meier method was used for survival analysis. Univariable Cox proportional hazard regression was used to identify factors significantly associated with the risk of death for all causes. Multivariable Cox proportional hazards regression analysis was used to determine independent significant factors associated with the risk of death for all causes, and hazard ratios (HR) and confidence intervals (CI) were calculated. Insurance status, income and education levels for each patient’s area of residence were also adjusted in the multivariable analysis. Results are based on adjusted variables. A *p*-value ≤ 0.05 was considered statistically significant. All statistical analyses were performed using SAS Version 9.4 statistical software, (SAS Institute Inc., Cary, NC, U.S.A., 2013).

## Results

**Fig. 1:**
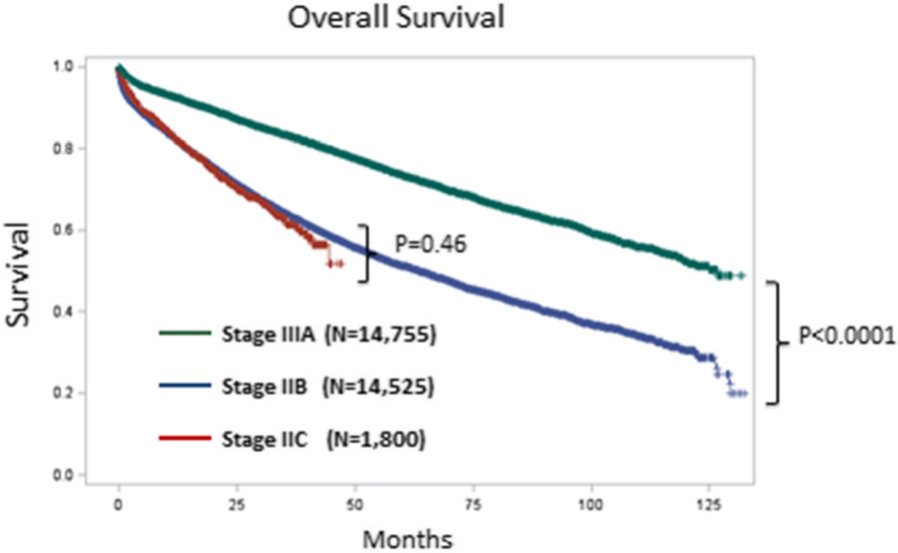
Overall Survival for Stage IIB/C and Stage IIIA: Note that there is a statistically significant survival difference between stage IIIA and stage IIB/C (*P* < 0.0001). However, there is no significant difference between stage IIB and stage IIC (*P* = 0.46)

**Fig. 2:**
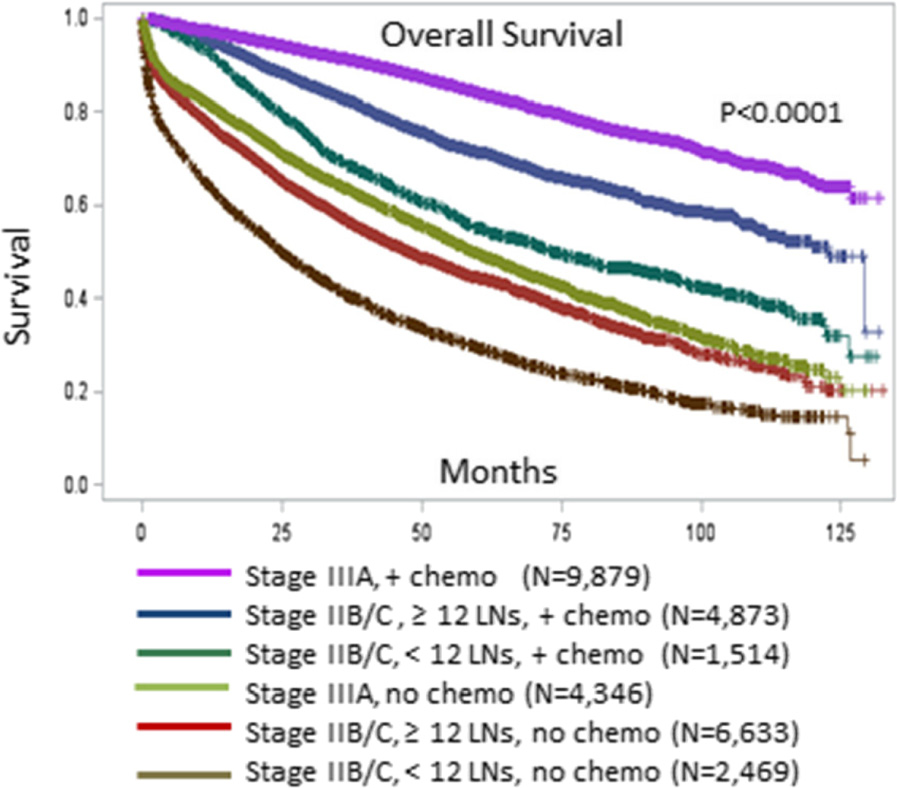
Overall Survival for the Six Subgroups of Patients with Stage IIB/C and Stage IIIA Colon Cancer. The 5-year OS are 84.1 % for stage IIIA plus chemotherapy, 70.8 % for stage IIB/C with ≥ 12 LNs plus chemotherapy, 53.9 % for stage IIB/C < 12 LNs plus chemotherapy, 49.5 % for stage IIIA without chemotherapy, 43.7 % for stage IIB/C ≥ 12 LNs without chemotherapy, and 27.7 % for stage IIB/C < 12 LNs without chemotherapy (*P* < 0.0001). The median survival has not been reached by the end of follow up (132 months) for stage IIIA with chemotherapy, 122.6 months for stage IIB/C, ≥ 12 LNs with chemotherapy, 72.5 months for stage IIB/C, < 12 LNs with chemotherapy, 58.9 months for stage IIIA without chemotherapy, 46.5 months for stage IIB/C, ≥ 12 LNs without chemotherapy, and 23.0 months for stage IIB/C, < 12 LNs without chemotherapy

The median follow-up was 39 months. Figure 1 demonstrates the Kaplan-Meier OS curve for stage IIB, stage IIC, and stage IIIA. Note that there is a significant survival difference between stage IIB/C and stage IIIA (*P* < 0.0001), although there was no significant survival difference between stage IIB and stage IIC (*P* = 0.46). Figure 2 demonstrates the Kaplan-Meier OS curve for the 6 subgroups which were defined by the number lymph nodes retrieved (<12 LNs vs ≥12 LNs) and whether or not systemic chemotherapy was given. For the entire cohort, the 5-year OS rate was 73.5 % for stage IIIA and 51.1 % for stage IIB/C. For the subgroups, the 5-year OS are 84.1 % for stage IIIA plus chemotherapy, 70.8 % for stage IIB/C with ≥ 12 LNs plus chemotherapy, 53.9 % for stage IIB/C < 12 LNs plus chemotherapy, 49.5 % for stage IIIA without chemotherapy, 43.7 % for stage IIB/C ≥ 12 LNs without chemotherapy, and 27.7 % for stage IIB/C < 12 LNs without chemotherapy (*P* < 0.0001). The median survival has not been reached by the end of follow up (132 months) for stage IIIA with chemotherapy; it was 122.6 months for stage IIB/C, ≥ 12 LNs with chemotherapy, 72.5 months for stage IIB/C, < 12 LNs with chemotherapy, 58.9 months for stage IIIA without chemotherapy, 46.5 months for stage IIB/C, ≥ 12 LNs without chemotherapy, and 23.0 months for stage IIB/C, < 12 LNs without chemotherapy.

The poorest survival subgroup was stage IIB/C with < 12 LNs retrieved and without receipt of adjuvant chemotherapy. Note that even when patients with stage IIB/C received optimal treatment (≥12 LNs retrieved and receipt of chemotherapy), their OS remains significantly lower than those with stage IIIA who had chemotherapy (Fig. 2).

Table 1 compares the demographic and therapeutic characteristics of stage IIB/C and stage III A before and after matching. Note that the two groups were fairly balanced after matching. Table 2 is a univariable analysis of factors associated with survival and Table 3 is the multivariable analysis based on the adjusted matched analysis. Note that stage IIB/C is an independent predictor of worse outcome compared to stage IIIA. T-stage and N-stage were not included because they were co-linear with stage of disease. Other independent factors associated with high hazard ratio include less than 12 lymph nodes retrieved, lack of receipt of chemotherapy, and positive margins. Before including LN dissection and chemotherapy in the multivariable model, the HR of stage IIB/C versus stage IIIA was 1.76 (95 % CI: 1.68–1.85). After adjusting for LN dissection and chemotherapy in addition to the adjustment of clinical variables included in Table 3, the HR was reduced to 1.51 (95 % CI: 1.44–1.59). Additional adjustment of demographic variables in Table 3 did not change the HR much (HR = 1.52; 95 % CI: 1.44–1.60).

**Table 1:** Comparison of demographic and therapeutic characteristics of stage IIB/C and stage IIIA

	Before matching			After matching		
Variable	Stage IIB/C	Stage IIIA	*P*	Stage IIB/C	Stage IIIA	*P*
No. of patients (%)	18,609 (53.2)	16,390 (46.8)		11,409 (50.0)	11,409 (50.0)	
Age			<.0001			0.94
18–49	1,678 (9.0)	1424 (8.7)		935 (8.2)	920 (8.1)	
50–64	4,628 (24.9)	5284 (32.2)		2,979 (26.1)	3,016 (26.4)	
65–74	4,368 (23.5)	4419 (27.0)		2,957 (25.9)	2,956 (25.9)	
75 and more	7,935 (42.6)	5263 (32.1)		4,538 (39.8)	4,517 (39.6)	
Distance from cancer reporting facility			0.06			0.58
<50 miles	16,909 (92.9)	15,015 (93.4)		10,630 (93.2)	10,651 (93.4)	
≥50 miles	1,303 (7.2)	1,066 (6.6)		779 (6.8)	758 (6.6)	
Race/Ethnicity			<.0001			0.93
White	15,964 (86.5)	13,450 (82.8)		9,785 (85.8)	9,770 (85.6)	
Black	1,965 (10.6)	2,197 (13.5)		1,283 (11.3)	1,308 (11.5)	
American Indian, Aleutian, or Eskimo	53 (0.3)	36 (0.2)		27 (0.2)	31 (0.3)	
Asian or Pacific Islander	390 (2.1)	456 (2.8)		261 (2.3)	251 (2.2)	
Other	89 (0.5)	100 (0.6)		53 (0.5)	49 (0.4)	
Facility Type			0.02			0.90
Community cancer program	2,715 (14.6)	2,239 (13.7)		1,565 (13.7)	1,588 (13.9)	
Comprehensive community cancer center	11,044 (59.4)	9,700 (59.2)		6,815 (59.7)	6,804 (59.6)	
Academic research program	4,825 (25.9)	4,432 (27.0)		3,029 (26.6)	3,017 (26.4)	
Other specified types of cancer program	25 (0.1)	19 (0.1)				
Facility Location			<.0001			0.99
New England	1,335 (7.2)	886 (5.4)		678 (5.9)	692 (6.1)	
Mid Atlantic	2,971 (16.0)	2,402 (14.7)		1,814 (15.9)	1,809 (15.9)	
South Atlantic	3,835 (20.6)	3,715 (22.7)		2,352 (20.6)	2,391 (21.0)	
East North Central	3,580 (19.2)	3,127 (19.1)		2,174 (19.1)	2,201 (19.3)	
East South Central	1,162 (6.2)	1,186 (7.2)		788 (6.9)	786 (6.9)	
West North Central	1,555 (8.4)	1,333 (8.1)		1,035 (9.1)	1,002 (8.8)	
West South Central	1,487 (8.0)	1,391 (8.5)		942 (8.3)	938 (8.2)	
Mountain	779 (4.2)	678 (4.1)		440 (3.9)	438 (3.8)	
Pacific	1,905 (10.2)	1,672 (10.2)		1,186 (10.4)	1,152 (10.1)	
Urban/Rural Location			0.05			0.96
Metro ≥1 million	9,488 (53.0)	8,380 (53.1)		6,039 (52.9)	6,028 (52.8)	
Metro 250 k to 1 million	3,863 (21.6)	3,556 (22.5)		2,539 (22.3)	2,554 (22.4)	
Urban <250 k	1,766 (9.9)	1,516 (9.6)		1,104 (9.7)	1,112 (9.8)	
Urban ≥ 20 k adjacent metro	731 (4.1)	657 (4.2)		460 (4.0)	476 (4.2)	
Urban ≥ 20 k not adjacent metro	262 (1.5)	221 (1.4)		182 (1.6)	175 (1.5)	
Urban <20 k adjacent metro	957 (5.4)	751 (4.8)		577 (5.1)	572 (5.0)	
Urban <20 k not adjacent metro	454 (2.5)	387 (2.5)		286 (2.5)	258 (2.3)	
Rural <2500 adjacent metro	164 (0.9)	169 (1.1)		108 (1.0)	109 (1.0)	
Rural <2500 not adjacent metro	212 (1.2)	154 (1.0)		114 (1.0)	125 (1.1)	
Comorbidities			<.0001			0.61
0	12,872 (69.2)	11,665 (71.2)		7,905 (69.3)	7,962 (69.8)	
1	4,185 (22.5)	3,537 (21.6)		2,576 (22.6)	2,513 (22.0)	
2	1,552 (8.3)	1,188 (7.3)		928 (8.1)	934 (8.2)	
Primary Site			<.0001			0.99
Cecum	4,917 (26.4)	3,941 (24.1)		3,077 (27.0)	3,077 (27.0)	
Ascending Colon	2,876 (15.5)	3,140 (19.2)		1,969 (17.3)	1,992 (17.5)	
Hepatic Flexure	748 (4.0)	608 (3.7)		473 (4.2)	461 (4.0)	
Transverse Colon	2,061 (11.1)	1,154 (7.0)		1,031 (9.0)	991 (8.7)	
Splenic Flexure	786 (4.2)	366 (2.2)		312 (2.7)	327 (2.9)	
Descending Colon	1,161 (6.2)	960 (5.9)		705 (6.2)	706 (6.2)	
Sigmoid Colon	5,324 (28.6)	5,756 (35.1)		3,524 (30.9)	3,535 (31.0)	
Overlapping Lesions	369 (2.0)	140 (0.9)		120 (1.1)	121 (1.1)	
Not Otherwise Specified	367 (2.0)	325 (2.0)		198 (1.7)	199 (1.7)	
Grade			<.0001			0.87
Well differentiated	1,506 (8.4)	1,779 (11.4)		1,087 (9.5)	1,084 (9.5)	
Moderately differentiated	11,868 (66.0)	11,226 (71.9)		8,007 (70.2)	8,049 (70.6)	
Poorly differentiated	4,004 (22.3)	2,381 (15.3)		2,084 (18.3)	2,059 (18.1)	
Undifferentiated, anaplastic	611 (3.4)	230 (1.5)		231 (2.0)	217 (1.9)	
Surgical Margins			<.0001			0.87
No residual tumor	14992 (82.6)	16,012 (98.8)		11,243 (98.6)	11,246 (98.6)	
With Residual tumor	3156 (17.4)	190 (1.2)		166 (1.5)	163 (1.4)	
Regional Lymph Nodes Examined			<.0001			<.0001
0–11	4,399 (23.8)	4,867 (29.9)		2,422 (21.3)	3,298 (29.0)	
12–90	14,101 (76.2)	11,434 (70.1)		8,944 (78.7)	8,078 (71.0)	
Chemotherapy			<.0001			<.0001
None	10,423 (58.7)	4,807 (30.5)		6,362 (58.4)	3,659 (33.3)	
Yes	7,335 (41.3)	10,975 (69.5)		4,525 (41.6)	7,327 (66.7)

**Table 2:** Univariable analysis of factors associated with overall survival

	Before matching			After matching		
Variable	HR	95 % CI	*P*-value	HR	95 % CI	*P*-value
Stage						
Stage IIB/C	2.15	(2.07, 2.24)	<.0001	1.70	(1.62, 1.78)	<.0001
Stage IIIA	1.00	–	–	1.00	–	–
Age						
18–49	1.00	–	–	1.00	–	–
50–64	1.30	(1.17, 1.45)	<.0001	1.41	(1.22, 1.63)	<.0001
65–74	2.23	(2.02, 2.48)	<.0001	2.48	(2.16, 2.86)	<.0001
75 and more	4.86	(4.41, 5.37)	<.0001	5.12	(4.47, 5.87)	<.0001
Race/ Ethnicity						
White	1.00	–	–	1.00	–	–
Black	0.96	(0.91, 1.02)	0.19	1.06	(0.98, 1.14)	0.14
American Indian, Aleutian, or Eskimo	0.90	(0.61, 1.33)	0.60	0.93	(0.58, 1.47)	0.75
Asian or Pacific Islander	0.55	(0.47, 0.64)	<.0001	0.62	(0.50, 0.75)	<.0001
Other	0.63	(0.46, 0.87)	0.005	0.64	(0.41, 0.99)	0.05
Facility Type						
Community cancer program	1.39	(1.31, 1.48)	<.0001	1.40	(1.30, 1.52)	<.0001
Comprehensive community cancer center	1.21	(1.16, 1.26)	<.0001	1.24	(1.17, 1.31)	<.0001
Academic research program	1.00	–	–	1.00	–	–
Facility Location						
New England	1.08	(0.99, 1.18)	0.10	0.94	(0.84, 1.06)	0.32
Mid Atlantic	1.07	(0.99, 1.16)	0.07	0.98	(0.89, 1.08)	0.68
South Atlantic	1.06	(0.99, 1.13)	0.12	1.00	(0.92, 1.09)	0.98
East North Central	1.10	(1.03, 1.18)	0.008	1.04	(0.95, 1.14)	0.39
East South Central	1.09	(0.998, 1.20)	0.05	1.08	(0.97, 1.21)	0.16
West North Central	1.10	(1.005, 1.19)	0.04	1.04	(0.94, 1.16)	0.43
West South Central	1.01	(0.92, 1.11)	0.80	0.99	(0.89, 1.11)	0.84
Mountain	0.93	(0.83, 1.04)	0.20	0.87	(0.75, 1.01)	0.06
Pacific	1.00	–	–	1.00	–	–
Urban/Rural Location						
Metro ≥1 million	1.00	–	–	1.00	–	–
Metro 250 k to 1 million	1.07	(1.02, 1.12)	0.008	1.09	(1.03, 1.15)	0.004
Urban <250 k	1.07	(1.004, 1.14)	0.04	1.06	(0.98, 1.14)	0.17
Urban ≥ 20 k adjacent metro	1.10	(1.002, 1.21)	0.046	1.10	(0.98, 1.24)	0.09
Urban ≥ 20 k not adjacent metro	1.04	(0.89, 1.22)	0.65	1.05	(0.87, 1.26)	0.63
Urban <20 k adjacent metro	1.13	(1.04, 1.23)	0.006	1.06	(0.95, 1.18)	0.30
Urban <20 k not adjacent metro	1.23	(1.10, 1.38)	0.0005	1.32	(1.15, 1.52)	0.0001
Rural <2500 adjacent metro	1.02	(0.85, 1.24)	0.81	0.88	(0.68, 1.14)	0.33
Rural <2500 not adjacent metro	1.12	(0.94, 1.33)	0.21	1.06	(0.85, 1.32)	0.58
Comorbidities						
0	1.00	–	–	1.00	–	–
1	1.46	(1.40, 1.53)	<.0001	1.49	(1.42, 1.58)	<.0001
2	2.29	(2.16, 2.42)	<.0001	2.29	(2.13, 2.46)	<.0001
Primary Site						
Cecum	1.00	–	–	1.00	–	–
Ascending Colon	0.96	(0.91, 1.02)	0.15	1.04	(0.97, 1.11)	0.30
Hepatic Flexure	0.97	(0.88, 1.07)	0.51	0.95	(0.84, 1.07)	0.39
Transverse Colon	1.07	(0.997, 1.14)	0.06	0.93	(0.85, 1.02)	0.11
Splenic Flexure	1.07	(0.96, 1.18)	0.23	0.90	(0.77, 1.04)	0.15
Descending Colon	0.85	(0.78, 0.93)	0.0003	0.85	(0.77, 0.95)	0.003
Sigmoid Colon	0.76	(0.72, 0.80)	<.0001	0.82	(0.77, 0.87)	<.0001
Overlapping Lesions	1.19	(1.03, 1.38)	0.02	0.80	(0.62, 1.03)	0.08
Not Otherwise Specified	1.14	(1.004, 1.29)	0.04	1.13	(0.95, 1.33)	0.17
Grade						
Well differentiated	1.00	–	–	1.00	–	–
Moderately differentiated	1.09	(1.02, 1.16)	0.01	1.04	(0.96, 1.13)	0.37
Poorly differentiated	1.39	(1.29, 1.50)	<.0001	1.11	(1.01, 1.22)	0.03
Undifferentiated, anaplastic	1.74	(1.53, 1.97)	<.0001	1.23	(1.02, 1.48)	0.03
Surgical Margins						
No residual tumor	1.00	–	–	1.00	–	–
With Residual tumor	1.91	(1.81, 2.02)	<.0001	1.24	(1.03, 1.49)	0.02
Readmission within 30 days of Surgery						
Not readmitted	1.00	–	–	1.00	–	–
Readmitted	1.15	(1.08, 1.22)	<.0001	1.13	(1.05, 1.22)	0.002
Regional Lymph Nodes Examined						
0–11	1.34	(1.29, 1.39)	<.0001	1.29	(1.23, 1.35)	<.0001
12–90	1.00	–	–	1.00	–	–
Chemotherapy						
Yes	1.00	–	–	1.00	–	–
No	3.55	(3.41, 3.70)	<.0001	3.49	(3.32, 3.67)	<.0001

**Table 3:** Multivariable analysis (After Matching) of factors associated with overall survival

Variable	HR	95 % CI	*P*-value
Stage			
Stage IIB/C	1.52	(1.44,1.60)	<.0001
Stage IIIA	1.00	–	–
Age			
18–49	–	–	–
50–64	1.24	(1.06, 1.44)	0.006
65–74	1.70	(1.44, 1.99)	<.0001
75 and more	2.85	(2.43, 3.35)	<.0001
Race/Ethnicity			
White	1.00	–	–
Black	1.18	(1.09, 1.28)	<.0001
American Indian, Aleutian, or Eskimo	1.15	(0.71, 1.87)	0.57
Asian or Pacific Islander	0.76	(0.62, 0.94)	0.01
Other	0.73	(0.45, 1.18)	0.20
Facility Type			
Community cancer program	1.29	(1.18, 1.40)	<.0001
Comprehensive community cancer center	1.15	(1.08, 1.22)	<.0001
Academic research program	1.00	–	–
Facility Location			
New England	1.00	(0.88, 1.14)	0.98
Mid Atlantic	0.99	(0.90, 1.10)	0.91
South Atlantic	0.95	(0.87, 1.05)	0.32
East North Central	0.95	(0.87, 1.05)	0.31
East South Central	0.98	(0.87, 1.11)	0.79
West North Central	1.02	(0.91, 1.15)	0.68
West South Central	0.96	(0.85, 1.08)	0.46
Mountain	0.91	(0.79, 1.06)	0.25
Pacific	1.00	–	–
Urban/Rural Location			
Metro ≥1 million	1.00	–	–
Metro 250 k to 1 million	1.02	(0.96, 1.08)	0.56
Urban <250 k	0.95	(0.87, 1.03)	0.22
Urban ≥ 20 k adjacent metro	0.98	(0.87, 1.11)	0.75
Urban ≥ 20 k not adjacent metro	0.89	(0.73, 1.08)	0.24
Urban <20 k adjacent metro	0.98	(0.87, 1.10)	0.68
Urban <20 k not adjacent metro	1.10	(0.94, 1.28)	0.25
Rural <2500 adjacent metro	0.93	(0.72, 1.21)	0.57
Rural <2500 not adjacent metro	0.90	(0.71, 1.14)	0.38
Comorbidities			
0	1.00	–	–
1	1.26	(1.19, 1.33)	<.0001
2	1.65	(1.53, 1.77)	<.0001
Primary Site			
Cecum	1.00	–	–
Ascending Colon	1.00	(0.93, 1.08)	0.99
Hepatic Flexure	1.02	(0.90, 1.16)	0.72
Transverse Colon	0.97	(0.88, 1.06)	0.45
Splenic Flexure	1.16	(0.99, 1.35)	0.07
Descending Colon	1.06	(0.95, 1.19)	0.31
Sigmoid Colon	0.98	(0.92, 1.05)	0.58
Overlapping Lesions	0.92	(0.70, 1.20)	0.54
Not Otherwise Specified	1.39	(1.17, 1.66)	0.0002
Readmission within 30 days of Surgery			
Not readmitted	1.00	–	–
Readmitted	1.21	(1.12, 1.31)	<.0001
Grade			
Well differentiated	1.00	–	–
Moderately differentiated	1.07	(0.98, 1.16)	0.12
Poorly differentiated	1.20	(1.09, 1.32)	0.0002
Undifferentiated, anaplastic	1.21	(1.00, 1.47)	0.05
Regional Lymph Nodes Examined			
0–11	1.30	(1.23, 1.37)	<.0001
12–90	1.00	–	–
Chemotherapy			
Yes	1.00	–	–
No	2.24	(2.11, 2.37)	<.0001
Surgical Margins			
No residual tumor	1.00	–	–
With Residual tumor	1.56	(1.29, 1.89)	0.003

## Discussion

Accurate cancer staging at the time of diagnosis assists clinicians to predict survival, impart prognostic information, and select the most effective treatments [10]. The last three decades, the American Joint Committee on Cancer (AJCC) has undergone multiple iterations. AJCC 5^th^ edition cancer staging system had only four categories for colon cancer, based on tumor-node-metastasis (TNM) classification (Stages I, II, III, IV) [11]. In 2002, AJCC 6^th^ edition subdivided stage II into IIA (T3N0) and IIB (T4N0) [12], but in 2000, the colorectal working group subdivided T4 into T4a (tumor penetrates the surface of the visceral peritoneum) and T4b (tumor directly invades or is histologically adherent to other organs or structures) [13] based on data that found that peritoneal involvement had an adverse outcome [14]. The latest AJCC 7^th^ edition published in 2010 further refined colorectal cancer staging by dividing N1 into N1a (metastasis in 1 node) and N1b (metastasis in 2–3 nodes), and N2 into N2a (metastasis in 4–6 nodes) and N2b (metastasis in ≥ 7 nodes). Consequently, stage II becomes IIA (T3N0), IIB (T4aN0), or IIC (T4bN0) and stage III becomes IIIA (T1-2 N1, T1N2a), IIIB (T3-4 N1, T2-3N2a, T1-2N2b), and IIIC (T4aN2a, T3-T4aN2b, T4bN1-2) [1].

Even with the latest iteration of AJCC, the OS for stage IIB/C remains lower than those with stage IIIA; the OS for stage IIB was 60.6 % versus 45.7 % for stage IIC and 67.2 to 73.7 % for stage IIIA [1]. Several groups attributed the survival paradox to either stage migration or lack of receipt of systemic therapy [2–6]. The implication is that if those with stage IIB/C received adequate lymph node dissection and adjuvant chemotherapy, their survival would have been better than those with stage III disease. No studies have demonstrated whether such is the case. Because the assumption has not been challenged, there is little impetus to revise the TNM staging system to account for the survival paradox. However, given our large robust database, we were able to analyze our cohorts based not only on receipt of chemotherapy, but also on the number of lymph nodes retrieved. To our knowledge, ours is the first and the largest dataset that assesses how these two factors, when combined, contribute to the paradox. Surprisingly, we found that even when adequate lymph nodes were retrieved and chemotherapy was administered, the survival paradox persists. What this suggests is that T4N0 lesions (i.e. stage IIB/C) may be inherently more aggressive than stage IIIA, although further research is necessary to delineate the root cause of the poorer survival among them.

Positive margins portend a poor outcome [15]. Inadequate resection of locally advanced lesions (i.e. T4) can lead to positive surgical margins, especially if an *en-bloc* resection was not performed [16]. In our study, positive margins impart a 56 % increased risk of death compared to negative margins. What impact surgical margins have on the paradox for optimally treated patients with stage IIB/C colon cancer will be the subject of our next investigation.

The role of adjuvant chemotherapy for patients with resected stage II colon cancer remains controversial. The 5-year OS following definitive colectomy for stage II colon cancer is in the range of 85-89 % [17]. Whether adjuvant systemic therapy can further improve this rate remains an area of intense investigation. Besides the QUASAR (Quick and Simple and Reliable) trial [18], which was a phase III trial that found OS benefit for patients with Stage II colon cancer, multiple other randomized trials as well as meta-analysis found no such significant advantage with chemotherapy [17, 19–25]. Although the commonly cited QUASAR trial (Quick and Simple and Reliable) showed an absolute improvement of 3.6 % for stage II colon cancer that received adjuvant therapy, the trial had several limitations [18]. Approximately 8.5 % of 3,239 patients who were thought to have stage II colon cancer actually had stage I or III disease and almost 30 % had rectal cancer (many of these patients received radiation therapy) [18]. To our knowledge, our analysis is the first to demonstrate a survival advantage of using adjuvant chemotherapy for stage II disease, but only for those with stage IIB/C colon cancer since we have 138,572 stage IIA patients in NCDB. Our data lend support to the American Society of Clinical Oncology’s recommendation of postoperative chemotherapy for patients with T4 tumors [26]. Of interest is that even when optimal treatment was rendered to those with stage IIB/C disease, their OS remains significantly lower than those with stage IIIA who had chemotherapy. Other factors that contribute to the survival paradox will need to be further investigated. Given our provocative data, we believe that randomized control studies are needed to determine whether there is a survival advantage of using adjuvant chemotherapy for stage II colon cancer.

One of the limitations of our study is the lack of recurrence data since this is not recorded in the NCDB. Additionally, we do not have specific causes of death and therefore it is plausible that deaths may not be related to cancer. We also do not have data on other factors such as microsatellite instability (MSI), preoperative CEA level, whether or not patients presented with obstruction or perforation, whether there was evidence of venous or perineural invasion, and whether blood transfusion had taken place, all of which are important prognosticators. However, our results are hypothesis-generating and can serve as a platform to further evaluate and explain the survival paradox.

## Conclusions

Based on our analysis of nearly 35,000 patients, we confirmed that a survival paradox between Stage IIIA and Stage IIB/C colon cancer patients exists for cases diagnosed in 2003–2012 in the ACoS’ hospitals. Although inadequate lymph nodes retrieved and lack of receipt of adjuvant chemotherapy contributes to stage IIB/C poor survival compared to stage IIIA, they themselves do not entirely explain the paradox. Further studies are necessary to determine other factors that also contribute to the paradox, including our hypothesis that stage IIB/C is inherently more aggressive than stage IIIA. Future iteration of AJCC staging of colon cancer should consider reconciling this paradox.

## Abbreviations

AJCC, American Joint Committee on Cancer; CEA, Carcinoembryogenic antigen; CI, Confidence interval; CoC, Commision of Cancer; HIPAA, Health Insurance Portability and Accountability Act; HR, Hazard ratio; IRB, Institutional Review Board; LN, lymph node; MSI, Microsatellite instability; NCDB, National Cancer Data Base; OS, overall survival; PUF, Participant User File; QUASAR, Quick and Simple and Reliable Trial
